# Editorial: Plant breeding innovations—CRISPR as a powerful weapon for agricultural crops

**DOI:** 10.3389/fgeed.2025.1623540

**Published:** 2025-05-22

**Authors:** Basavantraya N. Devanna, Yugander Arra, Maganti Sheshu Madhav

**Affiliations:** ^1^ Crop Improvement Division, Indian Council of Agricultural Research-Central Rice Research Institute, Cuttack, India; ^2^ Plant Production and Protection, Council for Scientific and Industrial Research-Central Institute of Medicinal and Aromatic Plants, Lucknow, India; ^3^ Plant Biotechnology, Academy of Scientific and Innovative Research (AcSIR), Ghaziabad, India; ^4^ Indian Council of Agricultural Research-National Institute for Research on Commercial Agriculture, Rajahmundry, India

**Keywords:** genome editing, CRISPR, plant breeding, CRISPRi, CRISPRa, prime editing, base editing, stress tolerance

## Introduction

The increasing global demand for food, accompanied by changing climatic scenarios, outbreaks of pests and diseases, soil degradation, deteriorating resources for agriculture, and the increasing demand for nutrient-rich food are significant present-day challenges for researchers and policymakers. The limited availability of desirable genetic mutations in plant germplasm and the lack of a precise tool to introduce such mutations have further augmented the challenges in crop breeding. The intensified challenges demand implementation of innovative plant breeding and improvement technologies to breed new crop varieties. One such pathbreaking technology is clustered regularly interspersed short palindromic repeats (CRISPR)-associated protein (Cas)-mediated genome editing. The first report on plant genome editing using CRISPR-Cas9 dates back as recently as 2013 (Li et al., 2013; Shan et al., 2013). Since then, CRISPR-Cas9 has taken a significant stride in the understanding of plant molecular biology, and genome editing applications using this technology have seen remarkable progress in plant breeding and varietal development programs. Furthermore, advancements in CRISPR technology, such as the recent prime-editing approach, have full potential to aid the plant breeder to opt for precise alterations in the genome to achieve desired changes without compromising the genetic content of already established and popular crop genotypes (Gupta et al., 2024).

In the frontiers in genome editing journal's research topic, we have compiled recent advances and applications of CRISPR-mediated genome editing with a focus on crop improvement. Five articles, including a research article on editing of the rice OsNAS2 gene promoter element have been published. Ludwig et al. used CRISPR/Cas9 to delete the cis-regulatory element (CRE) ARR1AT at position −933 in the promoter region of the rice *OsNAS2* gene for an enhanced per-plant accumulation of micronutrient Zn in the rice grain. In the context of efforts to improve food and nutritional security, the increased zinc (Zn) content in genome-edited rice lines holds significant potential to support global initiatives toward nutritional security as Zn is an essential micronutrient in human diets.

In one of the review articles in this Research Topic, Singh et al. emphasized the importance of CRISPR-Cas-mediated editing of the rice genome in creating novel alleles of known genes associated with the disease response. One suggested approach was altering the susceptibility genes without affecting the gene’s pleiotropic actions. This is important as knockout of some s-genes is known to have an adverse effect on rice growth and development. In rice, the impaired expression of *OsSWEET11a* and *OsSWEET11b* is known to induce male sterility and defective pollen development (Li et al., 2022). In addition, they also highlighted the regulation of host defense response through altering or modifying the key genes associated with general defense pathways in rice and their applications for enhanced defense response.

The beauty of CRISPR-Cas-mediated editing compared to similar techniques is the number of different CRISPR-based tools with particular applications, enabling researchers to attempt precise genome editing, including DNA and RNA editing. In one of the review articles, Bhuyan et al. have highlighted the variants of CRISPR-based tools such as CRISPR-Cas13-mediated RNA editing, CRISPR interference (CRISPRi), CRISPR activation (CRISPRa), base editors, prime editors, and CRISPR-guided caspase complex (CRASPASE). These CRISPR kits have the potential to make all possible kinds of desirable changes in rice to achieve desired changes, including resistance to fungal, bacterial, and viral diseases (Talakayala et al., 2022).

Although CRISPR-Cas-mediated genome editing is being successfully utilized for plant genome editing, the tools or the system are still evolving. Toda et al. have reviewed the recent developments in the field of approaches for plant genome editing using reproductive cells/tissues. One of the significant advantages of this approach, where the CRISPR-based editing system is directly delivered into reproductive cells such as pollen grains, zygotes, cells of embryos, and SAMs (shoot apical meristem), is heritable targeted mutagenesis, and also, this approach is out of the ambit of legislative concerns. Among the several approaches, controlled expression of exogenous Cas-gRNA complexes using reproductive cell-specific promoters, direct delivery of the editing system into pollen, delivery of the editing components into zygotes, and direct delivery of the CRISPR-Cas editing system into embryos and SAMs are some of the approaches. However, the availability of well-established protocols in most agriculturally essential crops is challenging to directly deliver CRISPR-Cas editing components into the reproductive cells or tissues (Reed and Bargmann, 2021).

Among the crops, rice is one of the most important and staple sources of food and nutrition for more than one-third of the global human population. CRISPR-Cas-mediated, new breeding technology is one of the latest tools at the disposal of rice breeders to address the present and emerging challenges in rice. Zafar et al. have systematically covered the applications of CRISPR-Cas-mediated genome editing in rice to address the merging difficulties with precision. Until now, more than 55 rice genes have been subjected to editing using the CRISPR-Cas approach for various traits such as abiotic and biotic stress tolerance, plant architecture, and grain yield (Rengasamy et al., 2024).

## Future prospects

The recent surge in genome crop editing indicates the promises this tool holds for the entire agriculture and society. As technology evolves, we also realize the challenges in its applications, specifically in crops. Notwithstanding the latest regulatory frameworks in several countries, the regulatory aspect of CRISPR-Cas-mediated genome editing is still a significant concern in most parts of the world. In addition to regulatory aspects, intellectual property (IP) issues governing the CRISPR, Cas, and relevant tools are the major bottlenecks in commercializing edited crops. Presently, there are thousands of patents, and it is very complex to understand and follow the licensing aspect of these patents. Along with these, the more researchable challenges include editing polyploid crops, manipulation of polygenic traits, limitations in addressing the genetic redundancy, and delivery and regeneration efficiency in some recalcitrant systems.
